# A novel therapeutic strategy for esophageal varices using endoscopic treatment combined with splenic artery embolization according to the Child-Pugh classification

**DOI:** 10.1371/journal.pone.0223153

**Published:** 2019-09-26

**Authors:** Tsuyoshi Ishikawa, Ryo Sasaki, Tatsuro Nishimura, Yuki Aibe, Issei Saeki, Takuya Iwamoto, Isao Hidaka, Taro Takami, Isao Sakaida

**Affiliations:** Yamaguchi University Graduate School of Medicine, Department of Gastroenterology & Hepatology, Ube-Yamaguchi, Japan; Cincinnati Children’s Hospital Medical Center, UNITED STATES

## Abstract

Variceal hemorrhage may cause high rebleeding and mortality rates. Preventing the first episode of variceal bleeding is mandatory in patients with high-risk esophageal varices (EV). This study aimed to identify factors that predict the recurrence of EV after endoscopic treatment (ET), and to develop a reasonable therapeutic strategy for EV in cirrhosis. From January 2012 to December 2014, 45 patients with cirrhosis and high-risk EV underwent ET, including sclerotherapy and/or ligation. Statistical analyses identified factors associated with the recurrence of EV after ET, and the Kaplan-Meier method determined the cumulative variceal recurrence rates. The 1-, 2-, and 3-year cumulative posttreatment recurrence rates for EV were 13.3%, 29.5%, and 32.2%, respectively. No significant differences were evident between the patients with and without variceal recurrences at 1-year posttreatment. The multivariate regression analyses identified a history of partial splenic embolization (PSE) and the pretreatment Child-Pugh classification as independent predictors of variceal recurrences at 2 years (*p* < 0.05) and 3 years (*p* < 0.05) posttreatment. While EV did not recur after ET and splenic artery embolization in cases with Child-Pugh class A, the overall posttreatment variceal recurrence rates were 0% and 66.7% when PSE was performed before and after ET, respectively, in those with Child-Pugh class B or C. Splenic artery embolization significantly reduced the hepatic venous pressure gradient and markedly lowered the Child-Pugh score in 15 patients. Adjunctive PSE and pretreatment Child-Pugh class A could be independently associated with reduced cumulative recurrence rates of EV post-ET. From the perspectives of portal hemodynamics and hepatic function, splenic artery embolization before or after ET could prevent posttreatment variceal recurrence in patients with Child-Pugh class A, and PSE before ET could achieve the long-term eradication of EV following ET in those with Child-Pugh class B or C.

## Introduction

Esophageal varices (EV) may be present in about 50% of patients with cirrhosis [1]. Since variceal hemorrhage may cause high rebleeding and mortality rates, preventing the first episode of variceal bleeding is mandatory in patients with high-risk EV. Endoscopic procedures, including endoscopic variceal ligation (EVL) and endoscopic injection sclerotherapy (EIS), are widely used to arrest and prevent bleeding. EVL is the gold standard treatment for variceal eradication, because of its unequivocal efficacy, greater convenience, and safety, and lower level of invasiveness compared with EIS. While EVL is plagued by high variceal recurrence rates caused by the mechanical effects of the rubber bands located within the submucosa, the chemical effect of sclerotherapy reaches the perforating veins and the paraesophageal collateral veins. The effectiveness of EIS combined with EVL, which augments the effect of the sclerosant on the deeper vessels and maintains the band ligation efficacy that quickly obliterates the varices, has been reported [2–6]. Hence, to reduce the recurrence of EV, combined therapy that comprises EIS and EVL, has been implemented as frequently as possible to treat EV prophylactically at our institute. However, despite using procedural techniques and follow-up systems of the same quality, a range of postoperative clinical courses have occurred, including the early recurrence or long-term eradication of EV, after endoscopic treatment (ET).

Some studies have tried to compare the variceal recurrence, rebleeding, and mortality rates after different ETs [2,5,7–14], but, to our knowledge, few studies have employed statistical analyses that have incorporated multiple clinical factors to predict the posttreatment recurrence of EV in patients with cirrhosis. This study aimed to identify predictive factors associated with variceal recurrence post-ET, and to develop a reasonable therapeutic strategy for EV in cirrhotic patients with portal hypertension.

## Materials and methods

### Study design and ethical considerations

This single-center, retrospective study involved examining patients’ medical records, and the laboratory data and imaging findings were reviewed. Each examination’s purpose was comprehensively explained, and informed consent was obtained from all of the participants in writing. The research was carried out in accordance with the Declaration of Helsinki, and was approved by the institutional review board of Yamaguchi University Hospital (H28-041).

### Patients

The study participants comprised patients with cirrhosis and high-risk EV who had undergone ET. The exclusion criteria were obstruction of the portal vein trunk, refractory ascites, and a Child-Pugh (CP) score ≥ 11. The biochemical, clinical, and ultrasonographic findings established a diagnosis of cirrhosis. Between January 2012 and December 2014, 65 patients with cirrhosis and EV underwent ET, comprising EIS and/or EVL, at our hospital. Nine patients with bleeding and 11 with variceal recurrences during the 3-year target period who were enrolled in this study were excluded from the analysis. Finally, data from 45 patients who underwent prophylactic ET, including EIS-based therapy, namely, EIS combined with EVL, and EVL alone, for EV were analyzed. Table 1 presents the patients’ baseline clinical characteristics.

**Table 1:** Patients’ baseline clinical characteristics (*n* = 45).

**Age, mean, years (SD; range)**	67.5 (8.4; 51–84)
**Sex, *n* (%)**	¤
**Male**	28 (62.2)
**Female**	17 (37.8)
**Etiology, *n* (%)**	¤
**HBV**	3 (6.7)
**HCV**	17 (37.8)
**Alcohol**	13 (28.9)
**NASH**	4 (8.9)
**Other**	8 (17.8)
**Child-Pugh score, mean (SD)**	6.5 (1.6)
**Class A, *n* (%)**	27 (60.0)
**Class B, *n* (%)**	17 (37.8)
**Class C, *n* (%)**	1 (2.2)
**Number of ETs, *n* (%)**	¤
**Initial treatment**	32 (71.1)
**Retreatment**	13 (28.9)
**Therapeutic procedure, *n* (%)**	¤
**EIS-based**	41 (91.1)
**EVL only**	4 (8.9)
**Form of EV, *n* (%)**	¤
**F1**	5 (11.1)
**F2**	34 (75.6)
**F3**	6 (13.3)
**RC signs on EV, *n* (%)**	¤
**RC0**	8 (17.8)
**RC1**	16 (35.6)
**RC2**	18 (40.0)
**RC3**	3 (6.7)
**History of PSE**	¤
**Present, *n* (%)**	15 (33.3)
**Before ET/after ET, *n*¤**	11/4¤
**Absent, *n* (%)**	30 (66.7)
**HCC**	¤
**Present, *n* (%)**	17 (37.8)
**Stage I/II/III/IV-A, *n*¤**	4/6/5/2¤
**Absent, *n* (%)**	28 (62.2)

SD, standard deviation; HBV, hepatitis B virus; HCV, hepatitis C virus; NASH, nonalcoholic steatohepatitis; ET, endoscopic treatment; EIS, endoscopic injection sclerotherapy; EVL, endoscopic variceal ligation; EV, esophageal varices; RC, red color; PSE, partial splenic embolization; HCC, hepatocellular carcinoma.

### Clinical and laboratory assessments

Hepatic function markers, including the total bilirubin and albumin levels, and the prothrombin time percentage activities, were evaluated within 3-days pre-ET, and the complete blood counts, including the white blood cell counts, hemoglobin concentrations, and platelet counts, and renal function, comprising the blood urea nitrogen and creatinine levels, were also evaluated. The hepatic functional reserve was assessed using the CP scoring and classification system, and renal function was assessed using the estimated glomerular filtration rate (eGFR). The hepatocellular carcinomas (HCCs) were staged using the Liver Cancer Study Group of Japan’s criteria and the following lesional characteristics: (1) solitary, (2) ≤ 2 cm wide, and (3) without vascular invasion [15]. Stage I lesions fulfilled all three criteria (T1), stage II lesions fulfilled two criteria (T2), stage III lesions fulfilled one criterion (T3), stage IV-A lesions did not fulfill any of the criteria (T4) and had no distant or lymph node metastases, and stage IV-B lesions did not fulfill any of the criteria and had distant metastases.

### Endoscopic evaluation of esophageal varices

The endoscopic findings from the EV were evaluated using *The general rules for study of portal hypertension* developed by the Japan Society for Portal Hypertension [16]. Hence, the EV were classified according to their color, namely, white or blue, form, namely, F1: straight, relatively small-caliber varices; F2: moderately enlarged, beady varices; or F3: markedly enlarged, nodular or tumor-shaped varices, and red color (RC) signs, including red wale marking, cherry red spots, and hematocystic spots, namely, RC1: a small number of RC signs in a limited location; RC2: between RC1 and RC3; or RC3: a large number of RC signs observed circumferentially. In Japan, F2 and F3 EV with RC signs indicate a high-risk of bleeding; therefore, prophylactic ET is generally performed on this high-risk group. Variceal eradication was defined as the nonvisualization of EV or the presence of only fibrosed varices that were resistant to ligation. Variceal recurrence was defined as the detection of F2, F3, or RC signs or ruptured EV, regardless of their form or RC signs, post-ET.

### Endoscopic variceal ligation and endoscopic injection sclerotherapy

Two expert accredited physicians performed the ET. Each physician determined each patient’s therapeutic strategy according to their hepatic and renal function; EIS-based therapy was chosen as frequently as possible.

EVL was performed using a standard endoscope attached to a pneumoactivated EVL device (Sumitomo Bakelite Co., Ltd., Tokyo, Japan), which was introduced along a flexible overtube (Sumitomo Bakelite Co., Ltd.). The varices were ligated sequentially from the most distal lesion. EVL was repeated weekly until the varices were completely eradicated; this usually required two or three sessions.

EIS was performed using a standard endoscope with a 6-cm oral side balloon (Create Medic Co., Ltd., Yokohama, Japan) and a 23- or 25-gauge injection needle (Top Co., Ltd., Tokyo, Japan). Under fluoroscopic guidance, intravariceal injections of 5% ethanolamine oleate (EO) (Oldamin; Aska Pharmaceutical Co., Ltd., Tokyo, Japan) were administered that filled the varices and the supplying vessels. The injections were repeated for multiple varices until the maximum amount of EO (0.4 mL/kg) was injected. Usually, sclerotherapy alone was performed weekly until the injectable varices had almost disappeared, then band ligation was undertaken at the injection sites during the final EIS session; this procedure was called EIS-based therapy, namely, EIS combined with EVL, in this study.

### Follow-up

After treatment, follow-up endoscopy was performed at 2–3 months, then every 6 months if recurrences and bleeding were absent. The study’s endpoints were variceal recurrence, including variceal rupture, death, or loss to follow-up. The follow-up period ended in December 2018.

### Partial splenic embolization

Two expert accredited physicians performed the partial splenic embolizations (PSEs). A patient’s therapeutic strategy was determined according to their platelet counts and spleen volume. In general, platelet counts < 5 × 10^4^/μL are thought to represent a high-risk of bleeding; therefore, at our institute, PSE is recommended for these high-risk patients with splenomegaly when platelet counts of < 5 × 10^4^/μL persist in three consecutive blood samples, regardless of the timing of invasive treatments, including ET for EV. In this study, all patients who met the indication for PSE and agreed with our recommendation underwent the PSE procedure.

PSE was performed using the “Takatsuka method” as described by Shimizu et al. [17]. Briefly, a percutaneous catheter was inserted into the right femoral artery under local anesthesia (1% lidocaine), and its tip was advanced into the splenic artery’s hilum. Gelatin sponges were implanted proximal to the microcoils that remained straight to embolize the splenic artery’s branches, and its upper branch remained untreated to achieve a final embolization rate of 70–80%. Contrast-enhanced computed tomography (CT) confirmed the infarct area at 1 week after PSE. Usually, no other treatments were performed, including ET for EV, for approximately 1-month post-PSE to enable the patients to recover physically.

### Wedged hepatic venous pressure and hepatic venous pressure gradient measurements

Before and immediately after PSE, the wedged hepatic venous pressure (WHVP) was measured and the hepatic venous pressure gradient (HVPG) was calculated, as described previously [18,19]. Briefly, the right hepatic venous branch was catheterized, and the free hepatic venous pressure and WHVP were measured using diluted contrast medium before and after vein occlusion, which was achieved by inflating a balloon catheter (Terumo Clinical Supply Co., Ltd., Gifu, Japan). The HVPG was defined as the pressure difference between the portal and hepatic veins, and was calculated by subtracting the free hepatic venous pressure from the WHVP.

### Statistical analyses

The statistical analyses were performed using JMP software, version 13 (SAS Institute Inc., Cary, NC, USA), and the data are expressed as the means and the standard deviations (SDs). The paired t-test was used for pairwise comparisons between the pre- and post-treatment data, and the unpaired t-test was used to compare two independent samples. The categorical variables were analyzed by using Fisher’s exact test. To identify factors that predicted variceal recurrence at 1-year, 2-years, and 3-years post-ET, univariate associations among the groups were assessed using the chi-squared test; this was followed by multivariate logistic regression analyses of the factors identified as significant (*p* < 0.05) by the univariate analyses to calculate the odds ratios, 95% confidence intervals, and *p* values. The cumulative variceal recurrence rates post-ET were plotted using the Kaplan-Meier method, and the significance of the differences was evaluated using the log-rank test. A value of *p* < 0.05 was considered statistically significant.

## Results

### Recurrence rates of esophageal varices after endoscopic treatment

**Fig 1:**
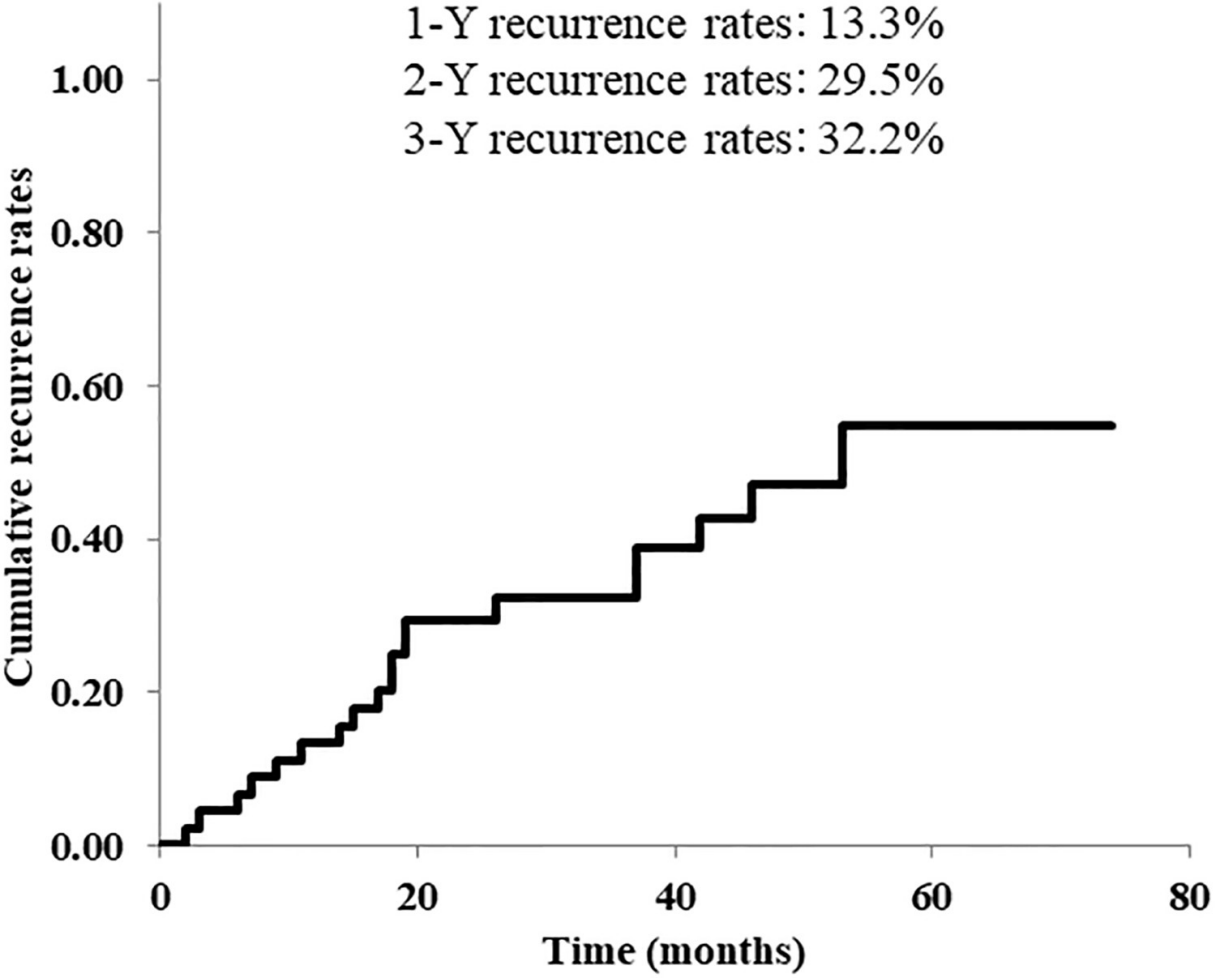
Cumulative recurrence rates of esophageal varices (EV) after endoscopic treatment (ET). During a median follow-up period of 30 months, recurrences of EV post-ET that required additional treatment occurred in 19 patients (42.2%), including seven patients with ruptured EV. After ET, the cumulative EV recurrence rates were 13.3% at 1 year, 29.5% at 2 years, and 32.2% at 3 years.

During a median follow-up period of 30 months, 19 patients (42.2%), including seven patients with ruptured EV, required additional treatment for EV recurrences after ET. The cumulative variceal recurrence rates at 1-year, 2-years, and 3-years posttreatment were 13.3%, 29.5%, and 32.2%, respectively, (Fig 1).

### Predictive factors for variceal recurrence after endoscopic treatment

No significant differences were evident between the patients with and without variceal recurrences at 1-year post-ET regarding age, sex, the cause of cirrhosis, the CP classification, the eGFR, the platelet count, the number of ETs, including initial treatment or retreatment, the therapeutic procedure, namely, EIS-based therapy or EVL alone, the EV form, the RC signs, a history of PSE, or concomitant HCCs. The univariate analyses showed that no history of PSE and pretreatment CP class B or C were significantly related to variceal recurrences at 2 years (*p* < 0.05) and 3 years (*p* < 0.05) posttreatment. The multivariate regression analyses identified a history of PSE and the pretreatment CP classification as independent predictors of variceal recurrences at 2 years (*p* < 0.05) and 3 years (*p* < 0.05) post-ET (Table 2).

**Table 2:** Univariate and multivariate analyses identifying factors that predict the recurrence of esophageal varices after endoscopic treatment.

¤	Univariate analysis			Multivariate analysis		
	Rec. (+)	Rec. (-)	*p* value	OR	95% CI	*p* value
**Two-year recurrence**	**¤**					
**CP classification** ** (A = 0/B or C = 1)¤**	4/9	19/7	0.010	23.8	2.3–245.4	0.015
**History of PSE** ** (Absence = 0/presence = 1)¤**	12/1	12/14	0.004	0.02	0.001–0.324	0.018
**Three-year recurrence**	¤					
**CP classification** **(A = 0/B or C = 1)¤**	5/9	15/5	0.022	16.3	1.6–161.6	0.027
**History of PSE** ** (Absence = 0/presence = 1)¤**	13/1	10/10	0.007	0.03	0.002–0.444	0.023

Rec, recurrence; OR, odds ratio; CI, confidence interval; CP, Child-Pugh; PSE, partial splenic embolization.

### Variceal recurrence according to the pretreatment Child-Pugh classification and/or a history of partial splenic embolization

**Fig 2:**
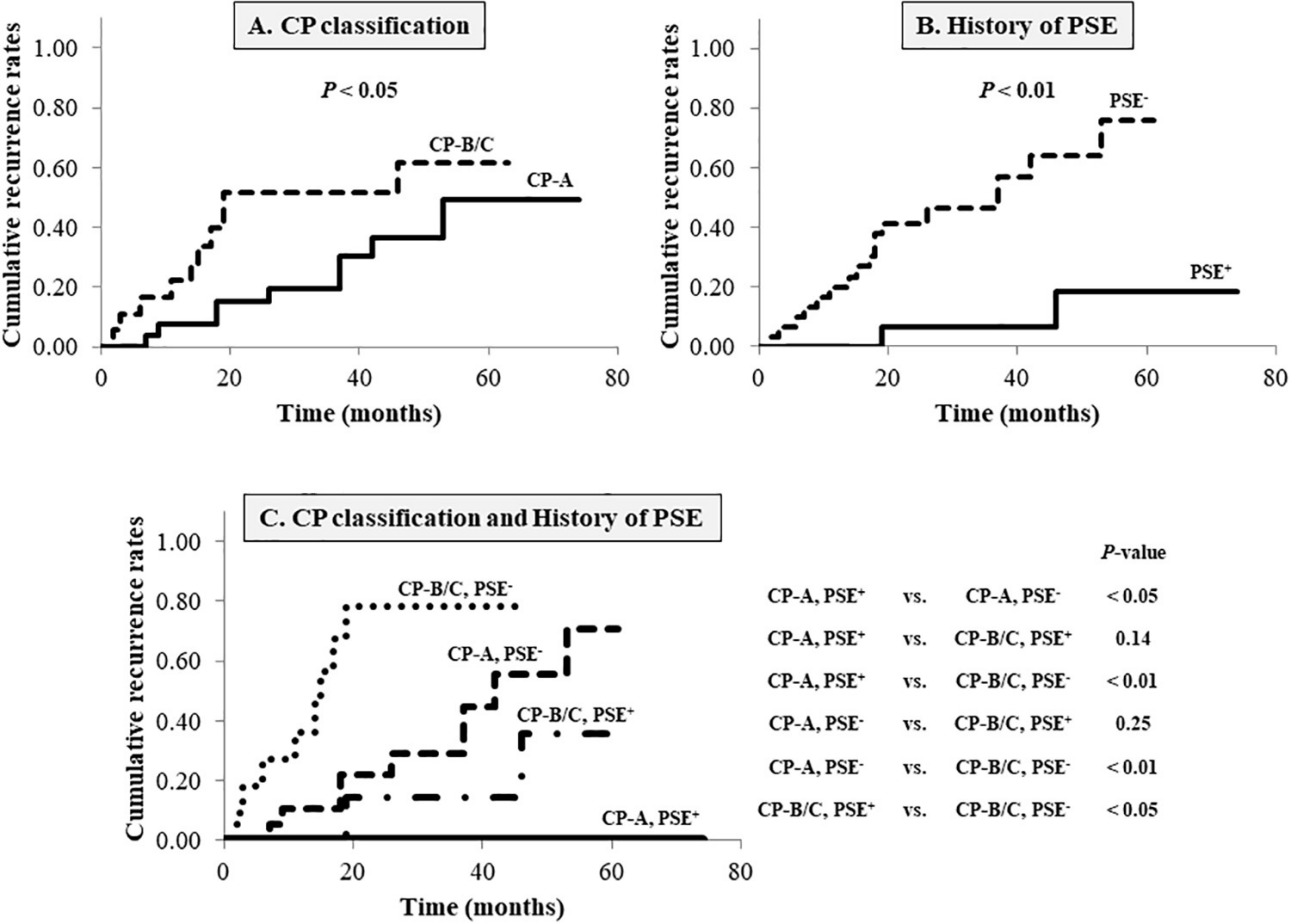
Cumulative recurrence rates of esophageal varices (EV) after endoscopic treatment (ET) according to the pretreatment Child-Pugh (CP) classification and/or a history of partial splenic embolization (PSE). (A), The cumulative recurrence rates of EV in the CP class A patients were significantly lower than those in the CP class B or C patients (*p* < 0.05). (B), ET combined with PSE significantly reduced posttreatment variceal recurrence compared with ET alone (*p* < 0.01). (C), During follow-up, there were no recurrences of EV after ET combined with PSE in the CP class A patients, and ET alone without PSE in the CP class B or C patients was associated with a significantly highest incidence of variceal recurrence. CP-A, Child-Pugh class A; CP-B/C, Child-Pugh class B or C; PSE, partial splenic embolization.

The cumulative posttreatment variceal recurrence rate was significantly lower for CP class A patients than that for CP class B or C patients (*p* < 0.05) (Fig 2A). Compared with ET alone, ET and PSE significantly reduced variceal recurrence (*p* < 0.01) (Fig 2B). No significant differences were evident between the CP class A and CP class B or C and the PSE^+^ and PSE^-^ subgroups regarding the pretreatment clinical characteristics. During follow-up, no variceal recurrences occurred after ET and PSE in the CP class A patients, and the CP class B or C patients who underwent ET alone without PSE had the highest EV recurrence rate post-ET (Fig 2C). Regardless of the CP classification, the cumulative posttreatment variceal recurrence rate was significantly lower in the patients who underwent ET and PSE compared with that in the patients who underwent ET alone (*p* < 0.05). Following PSE, no significant differences were apparent between the CP class A and CP class B or C groups regarding the cumulative recurrence rates of EV after treatment (*p* = 0.14). In the absence of PSE, the CP class A patients showed a significantly lower EV recurrence rate post-ET than the CP class B or C patients (*p* < 0.01). Table 3 presents the overall posttreatment variceal recurrence rates according to the timing of PSE and the pretreatment CP classifications of 15 patients. The median intervals between the two procedures were 1 month for 11 patients who underwent PSE before ET and 1 month for four patients who underwent PSE after ET. EV did not recur after treatment in the CP class A patients who underwent ET and PSE, regardless of the PSE timing. Among the CP class B or C patients, the posttreatment variceal recurrence rates when PSE was performed before and after ET were 0% and 66.7% (*p* = 0.06), respectively.

**Table 3:** Overall posttreatment recurrence rates of esophageal varices according to the timing of partial splenic embolization and the pretreatment Child-Pugh classification (*n* = 15).

	CP class A	CP class B or C	Total
**PSE before ET, % (*n*)**	0 (0/7)	0 (0/4)	0 (0/11)
**PSE after ET, % (*n*)**	0 (0/1)	66.7 (2/3)	50.0 (2/4)
**Total**	0 (0/8)	28.6 (2/7)	13.3 (2/15)

CP, Child-Pugh; PSE, partial splenic embolization; ET, endoscopic treatment.

### Effect of partial splenic embolization

**Fig 3:**
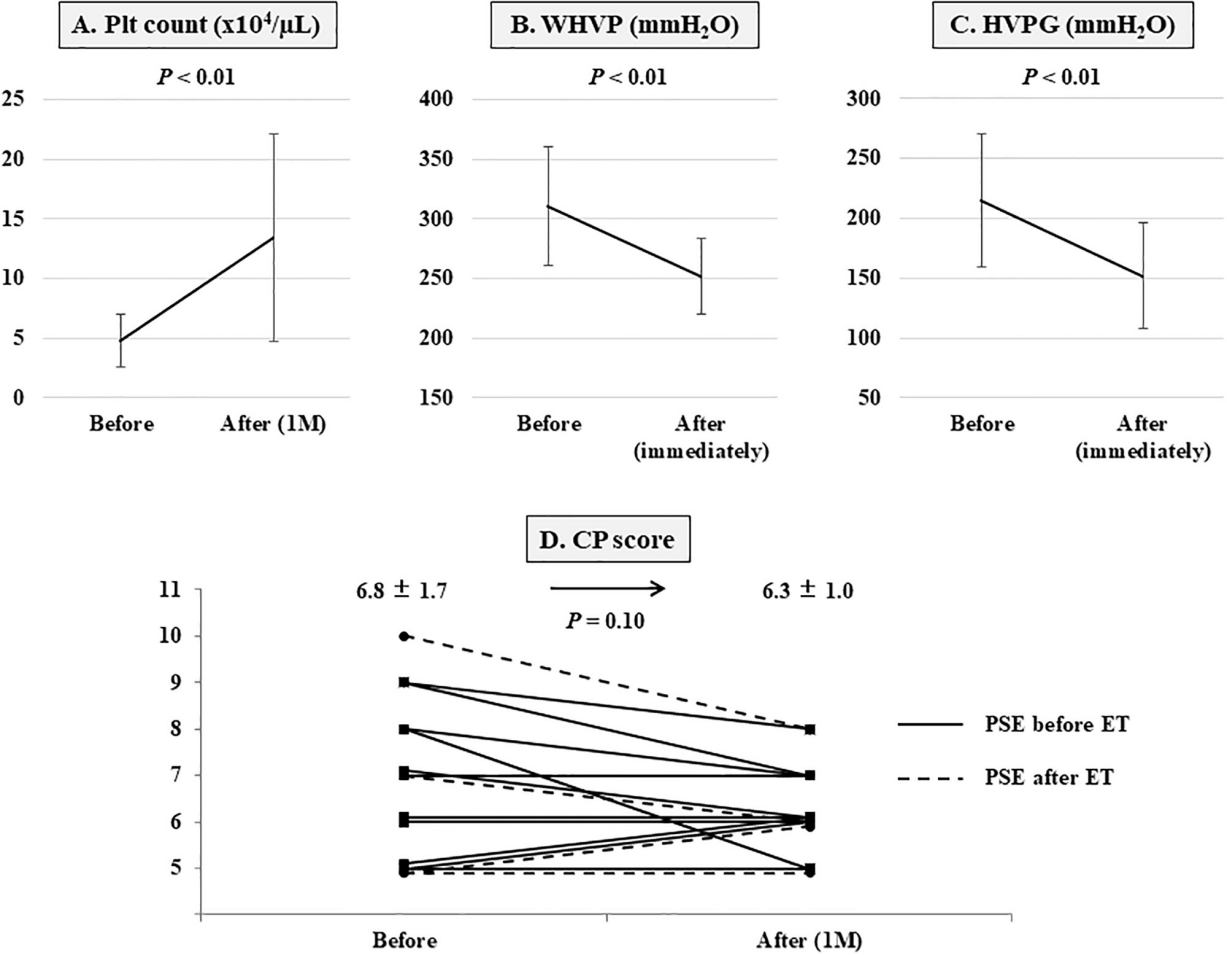
Hematological, hemodynamic, and hepatic functional changes in 15 patients who underwent partial splenic embolization (PSE) and endoscopic treatment. (A), The mean platelet count increased significantly from 4.8 × 10^4^/μL (standard deviation [SD] = 2.2) pre-PSE to 13.4 × 10^4^/μL (SD = 8.7) at 1-month post-PSE (*p* < 0.01). Splenic artery embolization significantly reduced the (B) mean wedged hepatic venous pressure from 310.5 mmH_2_O (SD = 49.4) to 251.5 mmH_2_O (SD = 31.8) (*p* < 0.01), and the (C) mean hepatic venous pressure gradient from 214.5 mmH_2_O (SD = 55.5) to 152.0 mmH_2_O (SD = 44.0) (*p* < 0.01), which were measured before and immediately after PSE. (D) The mean Child-Pugh (CP) score decreased from 6.8 (SD = 1.7) pre-PSE to 6.3 (SD = 1.0) at 1-month post-PSE (*p* = 0.10); the changes in the CP classifications at 1-month post-PSE were as follows: CP class A to CP class A (*n* = 7), CP class B to CP class A (*n* = 3), CP class B to CP class B (*n* = 4), and CP class C to CP class B (*n* = 1). Plt, platelet; WHVP, wedged hepatic venous pressure; HVPG, hepatic venous pressure gradient; CP, Child-Pugh; PSE, partial splenic embolization; ET, endoscopic treatment; 1M, 1 month.

The mean splenic embolization rate for the patients who underwent adjunctive PSE and ET (*n* = 15) was 75.4% (SD = 12.2); this was based on the contrast-enhanced CT findings at 1-week post-PSE. Splenic artery embolization significantly increased the mean platelet count from 4.8 × 10^4^/μL (SD = 2.2) pre-PSE to 13.4 × 10^4^/μL (SD = 8.7) at 1-month post-PSE (*p* < 0.01) (Fig 3A), and reduced the mean WHVP from 310.5 mmH_2_O (SD = 49.4) pre-PSE to 251.5 mmH_2_O (SD = 31.8) immediately post-PSE (*p* < 0.01) and the mean HVPG from 214.5 mmH_2_O (SD = 55.5) pre-PSE to 152.0 mmH_2_O (SD = 44.0) immediately post-PSE (*p* < 0.01) (Fig 3B and 3C). The mean CP score decreased from 6.8 (SD = 1.7) pre-PSE to 6.3 (SD = 1.0) at 1-month post-PSE (*p* = 0.10), and the CP classification changes at 1-month post-PSE were CP class A to CP class A (*n* = 7), CP class B to CP class A (*n* = 3), CP class B to CP class B (*n* = 4), and CP class C to CP class B (*n* = 1) (Fig 3D). Of these patients, two had portal thrombi and two had ascites, which were controlled by temporary medical treatment. In this study, no serious complications such as splenic abscess, pancreatitis, and pulmonary embolism were observed.

## Discussion

This study’s findings showed that a history of PSE and the pretreatment CP classification were independently associated with variceal recurrence post-ET. Previous studies’ findings have also shown that compared with EVL alone, EVL and PSE might prevent variceal recurrences and bleeding in cirrhotic patients [8,20,21]. Ohmoto et al. studied 84 patients with cirrhosis, large EV, and thrombocytopenia, and they showed lower rates of variceal development and hemorrhage in the patients who underwent EVL and PSE compared with those who underwent EVL alone [21]. While most studies have only compared variceal recurrence rates after EVL and PSE with those after EVL alone, our multivariate analyses identified a history of PSE as an independent predictor of variceal recurrence post-ET.

Although Spigos et al. [22] originally developed PSE for primary and secondary hypersplenism, its indications have become more wide-ranging following technical improvements [23,24]. PSE can amend not only hematological abnormalities, including thrombocytopenia and leukopenia, but also abnormal portal hemodynamics [25,26]. The HVPG and its changes may predict the development of new varices and variceal hemorrhage in patients with portal hypertension. In cirrhotic patients without esophagogastric varices, an HVPG decrease > 10% from baseline was associated with a reduction in the development of varices [27], and in cirrhotic patients with esophagogastric varices, an HVPG reduction to ≤ 12 mmHg, which is around 163 mmH_2_O or ≥ 20% compared with the baseline value, appeared to protect patients against variceal bleeding [28]. In our study, the mean pre- and post-PSE HVPG values were 214.5 mmH_2_O (SD = 55.5) and 152.0 mmH_2_O (SD = 44.0), respectively, which indicates that PSE reduced the HVPG to 152.0 mmH_2_O (< 163 mmH_2_O) and by 29.1% (> 20%). PSE might reduce the splenic and portal venous flows, and the subsequent portal venous pressure reduction may lower the likelihood of variceal recurrence. Thus, from the perspective of the portal-splenic vein hemodynamics, PSE is a useful adjunctive therapy to ET for high-risk EV.

The present study’s findings demonstrated that the pretreatment CP classification could predict variceal recurrence post-ET independently of PSE, which may be associated with differences in the degree of portal hypertension signified by the CP classification. Indeed, 72 patients’ HVPGs were measured at our institute, and the mean HVPG was significantly lower in the 46 CP class A patients (150.5 mmH_2_O [SD = 55.9]) than that in the 26 CP class B or C patients (179.6 mmH_2_O [SD = 57.9]) (*p* < 0.05), indicating that an HVPG < 163 mmH_2_O might be associated with better long-term control of EV following ET in CP class A patients. In addition, the mean total EO dose injected during EIS tended to be higher in the CP class A group (11.0 mL [SD = 7.1]) than that in the CP class B or C group (8.0 mL [SD = 5.8]) (*p* = 0.18), but there were no significant differences between the groups regarding the EV forms and RC signs; hence, more effective EIS could be performed on CP class A patients.

Incidentally, no studies have ever evaluated whether PSE should be performed before or after ET. Taniai et al. [8] performed PSE 1 week before treating EV prophylactically using EVL, and Xu et al. [20] performed PSE 1 week after successful hemostasis by the first EVL. In this study, EV did not recur in the CP class A patients who underwent ET and PSE, regardless of the timing of PSE. Furthermore, no variceal recurrences were observed when PSE was performed before ET on CP class B or C patients, but variceal recurrences occurred in 66.7% of the CP class B or C patients when PSE was performed after ET. Thus, the timing of splenic artery embolization requires attention, especially among CP class B or C patients. Notably, in this study, of the 11 patients who underwent PSE before ET, all five patients who were categorized as CP class A pre-PSE showed no changes in their CP classifications, but five (83.3%) and two (33.3%) of the six patients categorized as CP class B or C pre-PSE showed CP score decreases and improvements in the CP classification to class A, respectively, at 1-month post-PSE, which was similar to that seen pre-ET. If PSE can improve the CP classification from class B or C to class A, it could shift patients from the worst category, namely, CP class B or C without PSE that has the highest incidence of variceal recurrence, to the best category, namely, CP class A with PSE with no variceal recurrences. Hence, prior PSE could control hepatic function and improve patients’ clinical characteristics before they undergo ET for EV, thereby reducing posttreatment variceal recurrences, particularly among CP class B or C patients.

To the best of our knowledge, this is the first time a study’s findings have led to the proposal of a therapeutic strategy for EV in patients with cirrhosis that is based on the pretreatment CP classification and involves ET combined with splenic artery embolization, with a particular focus on the timing of PSE. However, the study’s results should be interpreted in the context of its limitations. First, this was a single-center retrospective study. Whether EVL or EIS is superior remains controversial [1,29,30], but EIS-based therapy, namely, EIS combined with EVL, has been mainly performed at our hospital for a variety of reasons, including posttreatment variceal recurrence and bleeding, and mortality. Consequently, only four patients (8.9%) in this study underwent EVL monotherapy, and the present study’s sample size was not sufficient to enable comparisons of the outcomes of ET according to the therapeutic procedures, namely, EIS-based therapy and EVL alone; therefore, much larger prospective studies are required to verify this study’s results. Second, although the applications of PSE are more wide-ranging, common indications for this procedure generally include hypersplenism with portal hypertension. Indeed, the primary indications of PSE at our institute are thrombocytopenia caused by hypersplenism. However, if patients with cirrhosis and high-risk varices do not have severe hypersplenism, could we perform PSE to reduce the portal venous pressure and improve hepatic function to prevent variceal recurrence following ET? If so, what are the appropriate splenic embolization rates in this situation? Thus, the indications for PSE as an adjunct to ET for EV have to be carefully reconsidered.

In conclusion, adjunctive PSE and a pretreatment CP class A could be independently associated with a reduced variceal recurrence rate post-ET. From the perspectives of portal hemodynamics and liver function, splenic artery embolization before or after ET could prevent posttreatment variceal recurrence in patients with CP class A, and PSE prior to ET would be reasonable for the long-term eradication of EV following ET in patients with CP class B or C.

## Supporting information

S1 File Minimal data set underlying the results. (XLSX)
